# Photo-Physical Characteristics of Janus Green B in Different Solvents and its Interaction Mechanism with Silver Nanoparticles

**DOI:** 10.1007/s10895-024-03723-8

**Published:** 2024-05-22

**Authors:** Sayed A. Abdel Gawad, R. Ghazy, S. Mansour, Hala Ahmed, Ahmed R. Ghazy

**Affiliations:** 1 Basic Science Center, Misr University for Science and Technology (MUST), 6 of October, Egypt; 2 Laser Laboratory, Physics Department, Faculty of Science, Tanta University, Tanta, 31527 Egypt

**Keywords:** Janus Green B, Quantum Yield, Photophysical Properties, Fluorescence, TD-DFT

## Abstract

This work explores the effects of solvent polarity on Janus Green B (JGB) photophysical properties. The Lippert-Mataga, Billot, and Ravi equations were utilized to calculate the singlet-state excited dipole moments (µ_e_) and ground state dipole moments (µ_g_) using absorption and fluorescence spectra analyses. The results showed an increase in the former, which is suggestive of electronic structural alterations upon excitation. Analysis of fluorescence quantum yield values revealed that JGB’s environment had an impact on its emission characteristics; it was particularly sensitive to silver nanoparticles, suggesting possible interactions. While simulations of electron density, electrostatic potential, and energy gap (E_g_) helped to understand the electronic structure of JGB, theoretical absorption spectra produced by Time Dependent Density Function Theory (TD-DFT) calculations offered insights into electronic transitions during absorption. To sum up, the present study contributes to our comprehension of the molecular behavior of JGB in various solvents by elucidating the intricate relationship among solvent polarity, molecular environment, and interactions with silver nanoparticles. Additionally, theoretical computations support the interpretation of experimental results.

## Introduction

Janus Green B (JGB), with the chemical formula Diethyl safranin-azo-dimethyl aniline, is a well-known member of the phenazine dye family. JGB, which can appear in a variety of hues from dark green to dark brown and black, needs to oxidize in order to show off its natural coloring abilities. Its unique ability to precisely label mitochondria in living cells makes it an invaluable tool for scientific research. It can also be used to stain sperm, yeast cells, chromosomes, nucleic acids, tissue culture monolayers, and a variety of tissues, including the brain, spinal cord, and fungus. Surprisingly, JGB has antimalarial effects when used medicinally, highlighting its diverse importance in biological research and medical applications [1, 2].

To fully utilize fluorescent materials, a thorough investigation of how environmental factors affect quantum yields and fluorescence spectra is necessary. Finding the electronically excited state’s dipole moment is a crucial first step towards understanding the transition state of a molecule’s electrical and geometrical structure. This knowledge is aided by computational methods that depend on solvatochromism-induced spectrum shifts. By observing variations in fluorescence and absorption maxima in liquids with different polarities, solvatochromism is clarified and offers detailed insights into molecule behavior in a variety of environments [3, 4].

A thorough understanding of the intrinsic photophysical features of laser dyes is crucial for developing active media for adjustable lasers [5]. These properties are controlled by solvent effects and external variables, particularly molecular structure. An essential component of this research is to determine the effects of a solvent on fluorescence and absorption bands, as well as molecular structural study using infrared (IR) spectra and bond length measurements [6]. The focus of this work is on solvent effects, which are separated into two groups: overall impacts and specific solute-solvent relationships. The study’s objective is to investigate the intricate dynamics of these interactions and clarify how they affect the chromophore’s optical properties.

As macroscopic features relevant to overall solvent effects, solvent polarity and polarizability are generated by solvating both the excited and ground states of the chromophore’s dipole moment [7, 8]. Solvent polarity parameters are widely discussed in literature. These include the Lippert parameter, which is related to the solvent’s refractive index and both the Dimroth Reichardt ET (30) parameter and the dielectric constant, which evaluates solvent polarizability utilizing the Kamlet-Abboud-Taft (π* scale) and solvatochromic dye spectroscopic data [9–12].

Solvatochromic effects are the result of solute-solvent interactions, which define a range of interactions between molecules, including charge-transfer multiplexes and hydrogen bonds. These interactions have a noteworthy effect on the optical features of the system. Analyzing these interactions provides important information on the dynamic interactions that shape solute molecule behavior in various solvent environments [13, 14]. Intramolecular charge transfer (ICT) distinguishes molecules having electron-accepting (A) and electron-donating (D) substituents. These molecules display unique optical and spectral characteristics. The importance of these molecules is shown by the appearance of large Variations in photophysical characteristics and redshifts in emission spectra in response to rising solvent polarity [15–17].

Investigating how noble metal nanoparticles affect photophysical processes in molecular media has received a lot of attention lately. Significant alterations in the radiative characteristics of noble metal nanoparticles are brought about when fluorophores are in close contact to their surface, which may boost luminescence. This discovery highlights the complex relationship between noble metal nanoparticles and fluorophores, providing information about possible improvements in the photophysical characteristics of nearby molecular systems [18, 19].

Janus Green B has attracted the researchers’ attention in recent years in different fields of study. Wu, Deng-Pan, et al. and Zhan, Jianhua, et al. studied the staining of mitochondria using Janus Green B [20, 21]. Keerthana, S. P., et al. studied the photocatalysis of Janus Green B using Sr doped TiO_2_ nanoparticles [22]. Wang, Huihui, et al. studied the application of Janus Green B in Flame-Retardant [23].

Even though Janus Green B has been studied extensively, especially in the areas of biological applications and mitochondrial staining, a thorough comprehension of its optical and photophysical properties is still elusive. By carefully analyzing Janus Green B’s behavior in a range of solvent conditions and using a variety of methods, such as Time-Dependent Density Functional Theory (TD-DFT) simulations, this work seeks to close this knowledge gap. Furthermore, the study investigates how fluorescence might be induced by interactions with silver nanoparticles, which contributes to our understanding of Janus Green B’s potential uses as a laser dye.

## Experimental

### Materials

Janus Geen B dye was purchased from Aldrich. (Empirical Formula: C_30_H_31_ClN_6_; Molecular Weight: 511.06; Dye content: 65%), the JGB solution (1 mM) was prepared. Rhodamine B (Empirical Formula: C_28_H_31_ClN_2_O_3_; Molecular Weight: 479.01; Dye content: 98%) Tetraethyl rhodamine Fluka *BioChemika*, for fluorescence.

The solvents used are **Ethanol** (CH_3_CH_2_OH) spectrophotometric grade (Aldrich), **Methanol** (CH_3_OH) spectrophotometric grade, ≥ 99% (Sigma), **Dimethyl Formamide** (HCON(CH_3_)_2_) for UV-spectroscopy, ACS reagent, ≥ 99.8% (GC) (Fluka), **Isopropanol**( C_3_H_7_OH) spectrophotometric grade, ≥ 99% (Sigma), **N-Methyl-2-pyrrolidone** (NMP) ,**Benzene** (C_6_H_6_**)** ACS spectrophotometric grade, ≥ 99% (Sigma- Aldrich), **Toluene** (C_6_H_5_CH_3_) puriss., absolute, over molecular sieve (H_2_O ≤ 0.005%), ≥ 99.7% (GC) (Fluka), **Diethyl Ether** ((CH_3_CH_2_)_2_O), purum, ≥ 99.0% (GC) (Fluka), **Cyclohexane** (C_6_H_12_) CHROMASOLV, for HPLC, ≥ 99.7% (Sigma- Aldrich), **Acetone** (CH_3_COCH_3_**)** ACS spectrophotometric grade, ≥ 99.5% (Sigma-Aldrich), **Ethylene Glycol** (HOCH_2_CH_2_OH**)** spectrophotometric grade, ≥ 99% (Aldrich) and **Dichloromethane** (CH_2_Cl_2_) for HPLC, ≥ 99.8% (GC) (Fluka). Other solvents such as **Distilled water** (H_2_O) and **Acetic acid** (**CH**_3_COOH) were supplied from Loba Chemie and Fisher companies. Every solvent used in this work was of spectroscopic grade, and its purity was previously examined for the presence of absorbing or fluorescent contaminants.

### Chemical Reduction Method for the Synthesis of Silver Nanoparticles

Silver nanoparticles (Ag NP) were created using a sodium borohydride (NaBH_4_) reduction technique. The reaction was started in an ice bath on a stir plate with constant stirring to control the breakdown of NaBH_4_. A 30 mL solution of 0.002 M NaBH_4_ was made in a flask. Laser beam reflection confirmed the colloidal nature of the solution when 2 mL of 0.001 M silver nitrate (AgNO_3_) was added at a regulated pace and immediately stopped stirring. For additional analysis, a tiny portion was transferred to a test tube and the color changed from pale yellow to grey due to agglomeration caused by the addition of a 1.5 M sodium chloride (NaCl) solution. Another part of the solution was treated with 0.3% polyvinyl pyrrolidone (PVP) to stop aggregation, which kept the color stable even after adding NaCl. A well-defined, spherical Ag NPs with a radius of 15–22 nm was discovered during characterization. When combined with careful inspection, this synthesis method yields consistent and stable silver nanoparticles that may be used in a variety of applications, demonstrating its promise for controlled nanoparticle production [24].

### Instrumentation

A Shimadzu Rf 510 Spectrofluorometer with band pass of 10 nm with xenon lamp and spectral range from 200 to 900 nm used with rectangular quartz cell of dimensions 1 cm x1cm for measuring emission spectrum. A Shimadzu UV-160 A Spectrophotometer with band pass 5 nm and operational spectral range of 200–900 nm was employed to record the UV-visible absorption spectra.

## Results and Discussions

### TD-DFT Simulation of Janus Green B

**Fig. 1:**
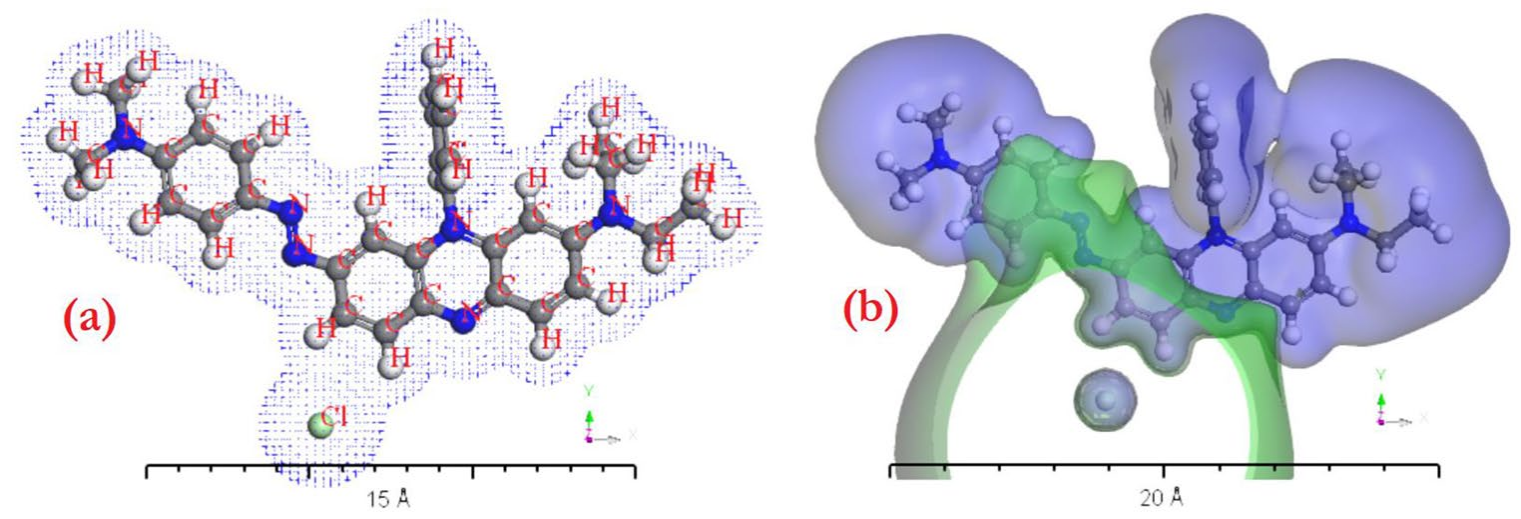
(a) Gas phase electron density of JGB, (b) the JGB gas phase’s electrostatic potential using TD-DFT/*Dmol*^*3*^ applications

The gaseous phase features of Janus Green B (JGB) were scrutinized through an examination of electron density and electrostatic potential. The optimization of JGB’s geometry was accomplished utilizing PW91’s DFT-DMOl^3^ computing algorithm. This study utilized electron density analysis to delve into the electron systems within the gaseous phase of JGB. Moreover, an exploration into potential extensions of the JGB gas phase was undertaken, showcasing potential diagrams. Comprehensive investigations of both electron density and electrostatic potential supported the inquiry into potential electron transport. JGB was analyzed in detail using these metrics in relation to the physical-chemical properties of its gaseous phase, as mentioned in [25, 26]. Further calculations could be performed utilizing Time-Dependent Density Functional Theory (TD-DFT) and TD-DFT/Gaussian ideas to compute the gaseous phase electron systems of JGB, as demonstrated by the electron density shown in Fig. 1(a and b). This multidisciplinary approach, which combines theoretical models with empirical data, provides a thorough knowledge of the behavior of JGB in its gaseous state.

**Fig. 2:**
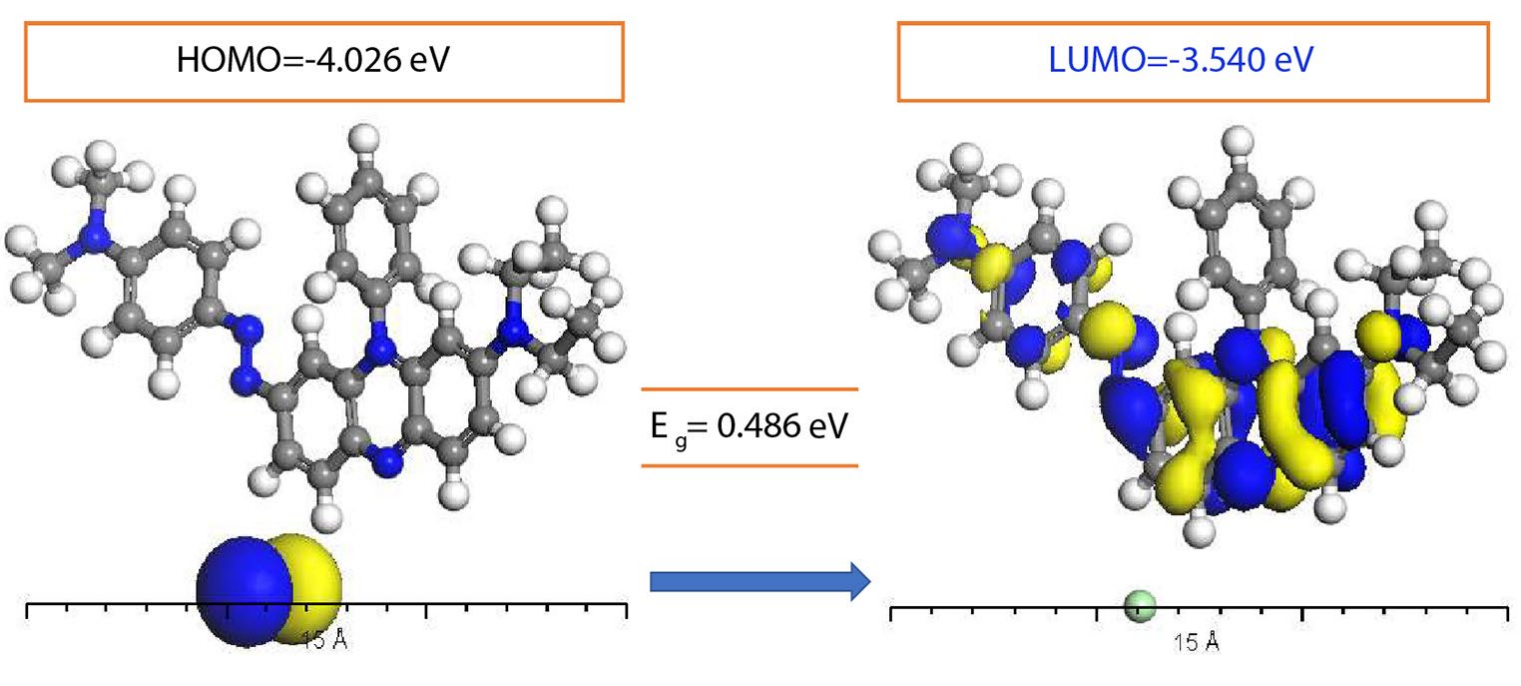
Absolute values of HOMO and LUMO states energy for JGB

With a focus on the difference between the lowest unoccupied molecular orbital (LUMO) and the highest occupied molecular orbital (HOMO), Fig. 2 illustrates the energy gap of the molecules as calculated using DFT-Dmol3. This computation sheds light on the molecules’ electronic structure by measuring the energy differential between their electron-filled and unoccupied orbitals. A thorough analysis of fragment molecular orbitals (FMOs) is made possible by the modelling of the HOMO and LUMO states of the molecules. This provides a thorough grasp of the molecular electronic configuration and its consequences for the system being studied.

Equations based on the values of the Highest Occupied Molecular Orbital (HOMO) and Lowest Unoccupied Molecular Orbital (LUMO) states energies can be used to easily calculate physio-chemical parameters that are essential for comprehending molecular properties, such as chemical potential (µ), softness (σ), global hardness (η), global softness (S), electronegativity (χ), global electrophilicity index (ω), and the maximum amount of electronic charge (ΔNmax). We have the following equations: $$\left( {\mu = \left( {{E_{HOMO}} + {E_{LUMO}}} \right)/2} \right),$$$$\left( {\eta = \left( {{E_{LUMO}} + {E_{HOMO}}} \right)/2} \right),$$$$\left( {\chi = - {\rm{\mu }}} \right),\,\left( {S = 1/2{\rm{\eta }}} \right),$$$$\left( {\omega = {\mu ^2}/2{\rm{\eta }}} \right),\,\left( {\sigma = 1/{\rm{\eta }}} \right)$$ and $$\left( {\Delta {N_{\max }} = - \mu /\eta } \right)$$ [27–29]. Together with the calculated values for µ, σ, S, η, χ, ω, and ΔNmax, Table 1 also shows the E_HOMO_ and E_LUMO_ values. When more electronic charge is added, the crucial quantum chemical property ω clarifies the molecule’s energy stability, and the molecule JGB is stable when E_HOMO_ and E_LUMO_ have negative values. These computed parameters provide important information about the behavior and molecular characteristics of the system being studied.

**Table 1:** Physio-chemical parameters for JGB as isolated molecules in different

Sample	E_HOMO_ (eV)	E_LUMO_ (eV)	E_g_^sim.^ (eV)	χ (eV)	µ (eV)	η (eV)	S (eV)	ω (eV)	$\varvec{\varDelta }{\varvec{N}}_{\varvec{m}\varvec{a}\varvec{x}}$	$\varvec{\sigma }$
JGB	-4.026	-3.540	0.486	3.783	-3.783	0.243	2.057	29.33	15.56	4.11

**Fig. 3:**
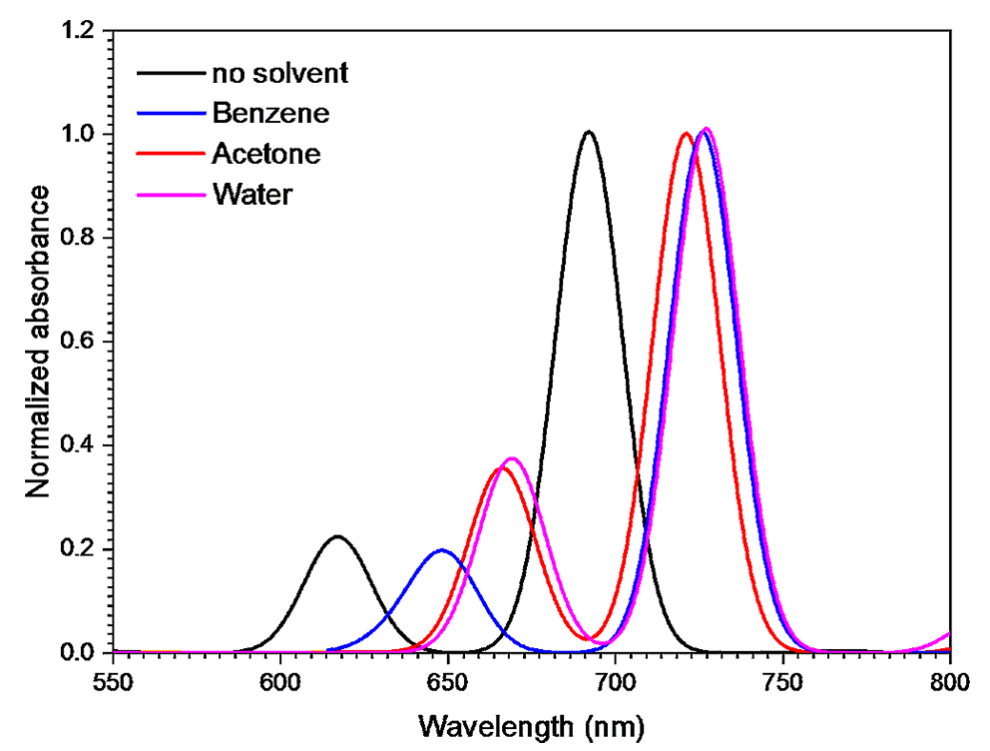
The simulated absorption spectra of JGB in different solvents using DFT-Dmol^3^ simulation

The effect of solvent polarity on the absorption spectrum of JGB was studied using optical properties in DFT-*Dmol*^*3*^ module. The absorption spectra of JGB dissolved in three different solvents (benzene, acetone and water) with different values of the dielectric constant (2.28, 20.7 and 78.54 respectively) is shown in Fig. 3. It is evident that the solvent polarity and the solute-solvent interaction have a significant influence on the dye’s absorption spectrum. Not only were new absorption bands visible, but the positions of the existing absorption bands were also changed.

### Impact of Solvents on the Absorption Spectra

**Fig. 4:**
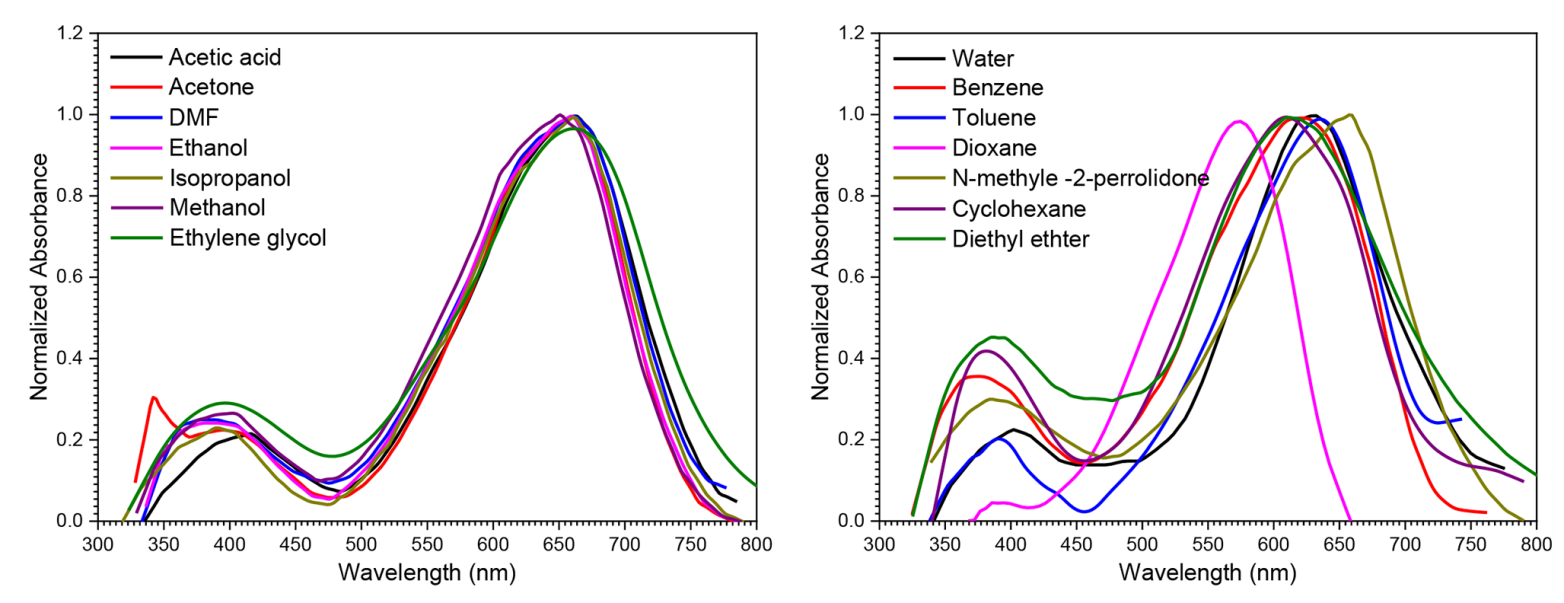
absorption spectra of 1 × 10^− 5^ mol dm^− 3^ of Janus Green B in different solvents

Figure 4 shows the UV-Vis absorption spectra of JGB at a concentration of 1 × 10^− 5^ mol dm^− 3^ in different solvents. Interestingly, variations in the solvent cause the absorption band’s position and form to fluctuate. The absorbance spectra of JGB in various solvents show that there is a major absorption band that is located between 577 and 666 nm, depending on the solvent. Furthermore, a secondary absorption band appears between 263 and 419 nm. These two different absorption bands for JGB indicate asymmetric protonation, which improves the molecule’s charge delocalization. This phenomena is consistent with observations made by Marino et al. [30].

The location of the absorption bands exhibits a red shift behavior when cyclohexane, a less polar solvent, is substituted for water, a more polar solvent. A bathochromic shift is specifically shown by the secondary band shifting from 381 nm to 396 nm and the main absorption band shifting from 605 nm to 629 nm. The interaction of the solvents with the nitrogen atom’s non-bonding electron pair of the dye is responsible for this behavior. The main reason for the observed spectrum shift is because in polar solvents, the solute-solvent interactions favorably maintain the π* orbital more steadily than the π orbital. This finding suggests that all of the compounds under investigation have greater polarity in their excited states than in their ground states. The ability of the compounds to give hydrogen and the solvents to receive hydrogen, as demonstrated by NMP and DMF, further demonstrates the red shift. A more stable excited state of the solute is indicated by positive solvatochromism (red shift), while a more stable ground electronic state relative to the excited state is indicated by a negative solvatochromism (blue shift) that results from an increase in solvent polarity [31].

**Fig. 5:**
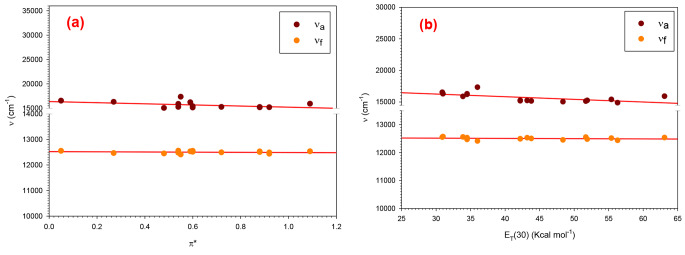
**(a)** Relationships between spectral properties in steady-state of Janus Green B, **(b)** Dependence of *v*_*abs*_ and *v*_*em*_ on the empirical solvent polarity parameter E_T_ (30) of Janus Green B

In Fig. 5a., data on steady state emission and absorption in a variety of solvents are displayed. The empirical π* scales established by Kamlet et al. are used to determine the solvent polarities [32, 33]. As a result of this property, hydrogen bonding effects in π* have been systematically eliminated as:

$${v_{abs}} = - 1152.3{{\rm{\pi }}^*} + 16365.5\left( {\;{\rm{c}}{{\rm{m}}^{ - 1}}} \right)\quad {r^2} = 27\%$$

$${v_{em}} = - 36.545{{\rm{\pi }}^*} + 12523.65\left( {\;{\rm{c}}{{\rm{m}}^{ - 1}}} \right)\quad {r^2} = 6\%$$

The empirical parameters, namely π*, α, and β, play a crucial role in gauging aspects such as solvent polarity, polarizability, hydrogen bond donor acidity, and hydrogen bond acceptor basicity. These parameters are intricately connected to absorption or emission data wavenumbers (ν) through the Taft and Kamlet equation, expressed as [34]:

$${\nu }_{max}={\nu }_{0}+s{\pi }^{\text{*}}+a\alpha +b\beta$$

In this equation, ν_o_ represents the regression intercept corresponding to the gaseous state of the spectrally active material. The coefficients s, a, and b are associated with the π*, α, and β parameters, respectively. ν_max_ signifies the wavenumber (cm^− 1^) at the peak of the absorption or emission band of Janus Green B (JGB) when dissolved in a pure solvent. This equation establishes a quantitative relationship between the unique molecular characteristics of the solvent and the specific spectral behavior of JGB, providing valuable insights into its interactions within diverse solvent environments. One could think of these coefficients as a solvatochromism measurement. For JGB dye absorption and emission, it may be like follows:

$${\nu }_{abs}=16480.77-1{152.3\pi }^{*}-1085.68\alpha -1650.84\beta \left({\text{c}\text{m}}^{-1}\right)$$

$${\nu }_{em}=12527.24-{ 36.545 \pi }^{*}-37.09\alpha -75.46\beta \left({\text{c}\text{m}}^{-1}\right)$$

The parameters known as solvatochromic (π*, α, and β) were obtained from published works [35, 36].

The wavenumber maximum *v*_*abs*_ and *v*_*em*_ graphs against the E_T_(30) parameter (also known as the Dimorth-Reichardt parameter) in Fig. 5b. illustrate how a solvent’s ionizing power (or, more accurately, its loose polarity) is based on the longest wavelength electronic absorption band’s maximum wavenumber, which is determined by the equation [37]:

$$\begin{gathered} {{\text{E}}_{\text{T}}}\left( {30} \right)=2.859 \times {10^{ - 3}}v \hfill \\ \,\,\,\,\,\,\,\,\,\,\,\,\,\,=2.859 \times {10^4}{\lambda ^{ - 1}} \hfill \\ \end{gathered}$$

where *v* is in cm^− 1^, ET (30) is in Kcal mol^− 1^, and λ is in nm.

A double linear correlation between the emission and absorption wave numbers was observed. An example of the improved solvent sensitivity *v*_*em*_ compared to *v*_*abs*_ would cause the dipole moment to rise upon excitation. In the excited state of the solvent, alcohols exhibit tighter hydrogen bonding aside from the dipolar interaction because of a higher charge density during excitation on the carbonyl oxygen. Therefore, the emission state of a protic solvent is a solvated complex that is rarely hydrogen-bonded and there is stabilization of charge separation as a result of excited state twisting intramolecular charge transfer (TICT) [38].

The oscillator strength (f), which indicates the number of electrons genuinely participating in the ground-to-excited state transition that gives rise to the absorption region in the electron spectrum, is a crucial characteristic of the dyes. Equation is used to calculate oscillator strength values [39]:

$$f=4.32 \times {10^{ - 9}}\int {\varepsilon \left( v \right)dv}$$

The molar extinction coefficient at wavenumber *v* is represented by *ε* (*v*).

The determination of oscillator strength values involved integrating the absorption spectra, and the results are presented in Table 2. Notably, it becomes evident from the table that the oscillator strength values exhibit an increase in solvents that display a higher propensity for electron redistribution. This phenomenon is attributed to the formation of hydrogen bonds with the two nitrogen atoms (-N = N-) positioned at the center of the molecule. Furthermore, the calculation and tabulation of the ground to excited state transition dipole moment (µ_12_) are detailed in Table 2, utilizing the equation [40]:

$${{\mu }^{2}}_{12}=\frac{f}{(4.72\times {10}^{7}\times {E}_{max})}$$

Here, E_max_ represents the maximum energy absorption. This equation provides a quantitative measure of the transition dipole moment, shedding light on the molecular dynamics during the transition from the ground to the excited state.

Fluorescence lifetime and non-radiative process rates are both significantly influenced by the fluorescence rate constant, sometimes referred to as the radiative decay rate constant (kr) for Janus Green B (JGB). It is directly correlated with the fluorescence resonance energy transfer (FRET) rate and the donor’s radiative decay rates. In the absence of non-radiative decay processes, the radiative (natural) lifetime (${\tau }_{0}$) of the excited state, which is the reciprocal of the fluorescence rate constant, provides a predictive metric for longevity. The radiative decay rate increases in more polarizable situations, indicating the clear sensitivity to the surrounding environment. The Strickler-Berg equation, derived from fundamental principles such as Einstein’s spontaneous emission rate and Planck’s black body radiation law, establishes a solid theoretical foundation for predicting the rate of a process. This equation is articulated as [41]:

$${k}_{r}=1/{\tau }_{0}=2.88\times {10}^{-9}{n}^{2}\frac{\int F\left(\nu \right)d\nu }{\int F\left(\nu \right){\nu }^{-3}d\nu }\int \frac{\epsilon \left(\nu \right)}{\nu }d\nu$$

In this expression, K_r_ denotes the rate constant, ${\tau }_{0}$ stands for the spontaneous emission lifetime, n represents the refractive index, ν is the wavenumber, F is the fluorescence intensity, and at a given wavenumber ν, the molar extinction coefficient is represented as ε(v). This equation offers a comprehensive approach for predicting the rate of the considered process, incorporating essential factors like fluorescence intensity, refractive index, and the molar extinction coefficient at specific wavenumbers.

The distance at which the original light intensity I_0_ reduced to (I = I_0_/e) known as the attenuation length (Λ) was also calculated using the equation [42]:

$${\Lambda }\left(\lambda \right)=\frac{1}{\epsilon \left(\lambda \right)cln\left(10\right)}$$

For each molar concentration c, and molar extinction coefficient ε(λ).

Additionally, the absorption cross-section ${\sigma }_{a}$ is calculated using the relation [43, 44]:

$${\sigma }_{a}=0.385\times {10}^{-20}\epsilon \left(\lambda \right)$$

**Table 2:** Absorption properties of Janus Green B in different solvents

Solvent	λ_abs_ (nm)	$\varvec{\epsilon }$ ×10^4^ M^− 1^ cm^− 1^	f	Λ cm	k_r_×10^8^ s^− 1^	sa ×10^− 16^ cm^2^	µ_12_ (D)
**Benzene**	618	2.62	0.51	1.66	1.53	1	15.23
**Acetic acid**	661	3.34	0.57	1.3	1.5	1.29	16.6
**DMF**	660	3.43	0.6	1.27	1.74	1.32	17.14
**Isopropanol**	666	3.78	0.64	1.15	1.7	1.46	17.68
**N-Methyle − 2-perrolidone**	645	4.6	0.89	0.94	6.98	1.78	20.51
**Acetone**	656	3.55	0.6	1.22	1.55	1.37	17
**Ethanol**	656	3.37	0.58	1.29	1.5	1.3	16.66
**Methanol**	650	3.55	0.61	1.22	1.49	1.37	17.12
**Water**	629	2.6	0.47	1.67	1.15	1	14.82
**Toluene**	630	2.07	0.4	2.09	3.79	0.8	13.6
**Diethyl Ether**	614	1.3	0.29	3.35	0.88	0.5	11.43
**Ethylene Glycol**	672	2.23	0.41	1.95	2.34	0.86	14.18
**Cyclohexane**	605	0.32	0.06	13.5	0.38	0.12	5.21
**1,4 Dioxane**	577	24.6	0.5	0.18	2.3	9.47	14.52

### Emission Spectra

**Fig. 6:**
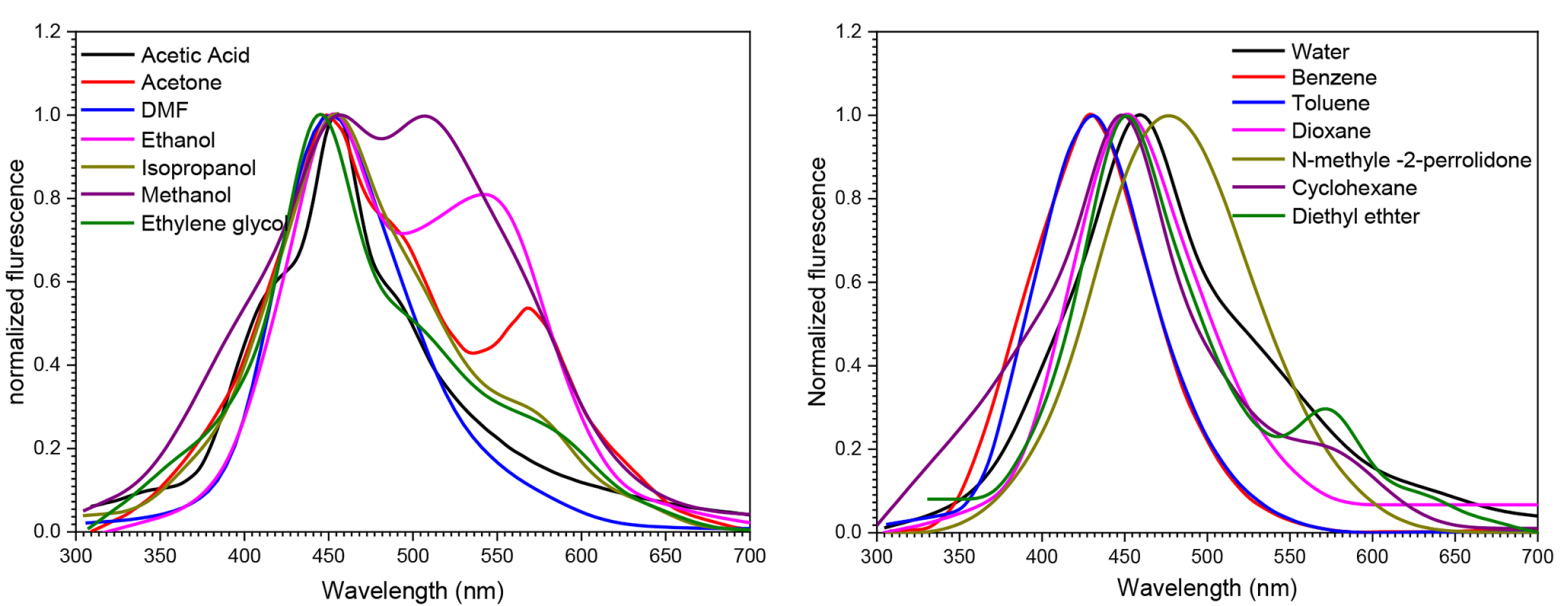
Emission spectra of 1 × 10^-5^ mol dm^-3^ of JGB in different solvents λ_ex_ = 295 nm

In Fig. 6, the emission spectrum of Janus Green B (JGB) in various solvents is presented, each with a concentration of 1 × 10^− 5^ mol dm^− 3^ and an excitation wavelength of 295 nm. It’s noteworthy that the experimental setup was designed to prevent the photodecomposition of JGB [45]. The emission spectrum reveals a prominent band within the 340–630 nm range. Notably, benzene exhibits the highest emission intensity, while water displays the lowest intensity. This observation is attributed to the increased radiative decay rate in benzene, as it favors radiative decay over nonradiative decay and quenching processes.

The emission characteristics of fluorophores typically manifest at longer wavelengths than those of absorption, resulting in what is known as the Stokes’ shift. The loss of energy in this shift can be attributed to various energetic processes. These include dissipation of energy due to electron redistribution within the solvent molecules in the immediate vicinity, influenced by the altered (usually increased) excited fluorophore’s dipole moment. Additionally, particular interactions between the solvent or solutes and the fluorophore as well as vibrational energy, include hydrogen bonding and the creation of charge-transfer complexes, contribute to the Stokes’ shift. Understanding these energetic processes provides insights into the emission behavior and characteristics of JGB in different solvent environments.

Table 3 show photo physical parameter such as relative fluorescence quantum yield (Ø_f_) which can be define as:

$${\varphi }_{f}=\frac{number \ of \ emmitted \ photond}{number \ of \ absorbed \ photons}$$

Use of the following connection yields the fluorophore’s quantum yield in relation to a standard material [46, 47]:

$${\varnothing }_{u}={\varnothing }_{s}\times \frac{{I}_{u}}{{I}_{s}}\times \frac{{A}_{s}}{{A}_{u}}\times \frac{{n}_{u}^{2}}{{n}_{s}^{2}}$$

In the case where Ø_s_ is the standard’s fluorescence quantum yield and Ø_u_ is the unknown’s, I_u_: The unknown’s area under the emission curve, I_s_: The standard’s area under the emission curve. A_u_: Unknown Absorbance A_s_: Standard Absorbance, n_u_: The solvent’s refractive index for the unknown, and n_s_: The solvent’s refractive index for the standard.

A standard reference, Rhodamine B in ethanol with a known quantum yield (ϕ_f_ = 0.7), was employed for the measurements. To maintain consistency, optical densities of both the sample and standard solutions were carefully controlled, always ensuring they remained below 0.1. The integrated intensities were determined within the wavelength range of 300 to 840 nm. Due to the choice of an excitation wavelength at 295 nm, extrapolation to the blue side of the spectra was deemed necessary for a comprehensive analysis of the data. This meticulous approach guarantees accurate and reliable measurements, aligning with established practices in spectroscopy [48]. The Fluorescence lifetime, emission cross-section, fluorescence energy yield, and intersystem crossing rate constant are comprehensively presented in Table 3, with calculations based on the following equations. The table serves as a repository for key parameters characterizing the fluorescence properties under investigation. The equations used for these calculations, though not explicitly stated, are instrumental in deriving these vital metrics, offering a detailed insight into the dynamic behavior of the system under study.

The equation can be utilized to ascertain the fluorescence lifespan (${\tau }_{f}$):

$${\tau }_{f}={\tau }_{0}{\varphi }_{f}$$

We substitute on ${\tau }_{0}$for the absorption band. The approximate connect between the intersystem crossing rate constant (k_isc_) and the fluorescence quantum yield Ø_f_ for (Ø_f_ =1) is [49]:

$${K}_{isc}=\left(1-{\varnothing }_{f}\right)/{\tau }_{f}$$

The energy yield of fluorescence (E_f_) estimated by [50]:

$${E}_{f}={\varphi }_{f}\frac{{\lambda }_{A}}{{\lambda }_{f}}$$

The following formula provides the emission cross-section σ_e_, a crucial indicator of laser dye quality [51]:

$${\sigma }_{e}=\frac{{\lambda }^{4}F\left(\lambda \right){\varnothing }_{f}}{8\pi c{n}^{2}{\tau }_{f}}$$

where c is the light’s velocity, λ is the emission wavelength, n is refractive index of solvent, and F(λ) is the normalized fluorescence spectrum since$\int F\left(\lambda \right)d\lambda =1$and ${\varphi }_{f}$is the fluorescence quantum yield.

For JGB we note that the low value of fluorescence lifetime and energy yield of fluorescence is due to the low fluorescence quantum yield of JGB.

**Table 3:** JGB’s fluorescence characteristics in various solvents at an excitation wavelength of 295 nm

Solvent	λ_emi_ (nm)	Ø_f_	τ_f_ (ns)	σ_e_ × 10^− 17^ cm^2^	E_f_ ×10^− 3^	k_isc_ ×10^10^
**Benzene**	434	0.13	0.903	2.87	113.8	0.096
**Acetic acid**	454	0.008	0.062	2.589	7.084	1.6
**DMF**	451	0.04	0.313	3.264	34.589	0.31
**isopropanol**	450	0.008	0.05	3.445	7.076	1.98
**N-mehyle − 2-perrolidone**	478	0.16	0.59	6.768	127.866	0.142
**Acetone**	449	0.008	0.081	44.02	6.895	1.22
**Ethanol**	454	0.008	0.062	2.56	6.978	1.6
**Methanol**	452	0.01	0.065	3.127	8.805	1.52
**water**	457	0.006	0.076	2.1	5.199	1.31
**Toluene**	431	0.34	0.692	9.188	240.6	0.0953
**Diethyl Ether**	451	0.06	1.57	0.371	51.2	0.0599
**Ethylene Glycol**	445	0.02	0.247	1.479	18.022	0.397
**Cyclohexane**	448	0.17	12.8	0.289	144.576	0.0065
**1,4 Dioxane**	453	0.05	0.031	31.81	29.029	3.06

### Dipole Moment Calculation

The influence of internal or external electric fields controls a molecule’s spectral band, which in turn controls the excited state dipole moment of the molecule. Solvatochromism appears as a potent tool for explaining these effects in the context of an internal electric field. The study of solubility—the ability of a molecule’s electronic absorption spectra to change in response to variations in the solvent environment around it, indicating changes in the molecule’s dipole moment—is known as solvatochromism.

In the context of solvatochromism, several particular approaches that depend on the internal electric field have been extensively utilized in this study. These methods explore the complex interactions between the electric field and the molecular structure, illuminating the subtleties of how solvents and their different polarity affect the electronic characteristics of the excited state molecule. Investigating these approaches helps us better understand the solvatochromic effects and offers insightful information about the broader implications for the dynamics of the examined molecule’s dipole moment.

### Lippert –Matage Method

The solvent dependence of absorption and fluorescence band maxima is a valuable approach for determining the variations in ground state and excited state dipole moments (µ_e_ – µ_g_) among different compounds. This difference can be quantified using the Lippert-Mataga equation, a useful tool in understanding the impact of solvents on molecular properties. The Lippert-Mataga equation allows for the calculation of the change in dipole moment by considering the shift in absorption and fluorescence maxima in different solvents, providing insights into the polarity of the molecules and their interactions with the surrounding environment. The equation forms a fundamental basis for studying the influence of solvents on the electronic structure and behavior of molecules [52, 53]:

$${\Delta }\nu ={\nu }_{abs}-{\nu }_{em}=\frac{{2\left({\mu }_{e}-{\mu }_{g}\right)}^{2}}{hca}{\Delta }f+constant$$

$${\Delta }f=\frac{D-1}{2D+1}-\frac{{n}^{2}-1}{2{n}^{2}+1}$$

where c is the speed of light in a vacuum and h is Planck’s constant. A is the cavity radius, D and n are the solvent’s dielectric and refractive indices, and µ_g_ and µ_e_ are ground and excited states dipole moments.

Suppan’s equation, which provides the molecular volume of molecules, was used to get the Onsager cavity radius a [54, 55]:

$$a=\sqrt[3]{\frac{3M}{4{\Pi }\rho N}}$$

Where M is the fluorophore molecular weight; $\rho$ is the fluorophore density, N is the Avogadro’s number. Where it is difficult to calculate the density of JGB we will suppose a = 4.5 Å, Lippert-Mataga equation can write in the following form:

$${\mu }_{e}-{\mu }_{g}=0.010{\left(m{a}^{3}\right)}^{1/2}$$

where m is the slope of the line (Stokes shift versus polarity Δf) in Cm^− 1^, µ in D (Deby), and a is given in Å [56]. Using the gaussian program, the ground state dipole moment µ_g_ was determined once the molecular geometry was entirely optimized. Table 4 provides a summary of the comparable data.

### Billot Method

By using the solvent polarity parameter:

$${\nu }_{abs}-{\nu }_{em}={m}_{1}\text{f}\left(D,n\right)+constant$$

$${\nu }_{abs}-{\nu }_{em}=-{m}_{2}(\text{f}\left(D,n\right)+2\text{g}\left(n\right))+constant$$

where the solvent polarity parameter, denoted by g(n) and f(D, n), is provided by [54, 57]:

$$\text{f}\left(D,n\right)=\frac{2{n}^{2}+1}{{n}^{2}+2}\left[\frac{D-1}{D+2}-\frac{{n}^{2}-1}{{n}^{2}+2}\right]$$

$$\text{g}\left(n\right)=\frac{3}{2}\left[\frac{{n}^{4}-1}{{(n+2)}^{2}}\right]$$

$${m}_{1}=\left(2{({\mu }_{e}-{\mu }_{g})}^{2}\right)/hc{a}^{3} \ {m}_{2}=\frac{2({{\mu }_{e}}^{2}-{{\mu }_{g}}^{2})}{hc{a}^{3}}$$

$${\mu }_{g}=0.01\frac{({m}_{2}-{m}_{1})}{2}{\left[\frac{{a}^{3}}{{m}_{1}}\right]}^{1/2}$$

$${\mu }_{e}=0.01\frac{({m}_{2}+{m}_{1})}{2}{\left[\frac{{a}^{3}}{{m}_{1}}\right]}^{1/2}$$

The first peak of absorption and emission’s stock shift was plotted against the functions f(D, n) and f(D, n) + 2 g(n), and the values of m_1_ and m_2_ were determined from the slope, which was used to calculate the dipole moment. Table 4 presents a summary of the appropriate data.

### Ravi Method

The evaluation of the Onsager radius (a) and the associated challenges have been mitigated by employing a ratio of two Onsager radii (aB / a), as highlighted by Ravi et al. [58]. The equation presented for measuring the excited state dipole moment is as follows:

$$\varDelta \nu =11,307\left[{\left(\frac{{\Delta }\mu }{{\Delta }{\mu }_{\text{{\rm B}}}}\right)}^{2}{\left(\frac{{a}_{\text{{\rm B}}}}{a}\right)}^{3}\right]{E}_{T}^{N}+constant$$

This equation provides a method for determining the excited state dipole moment, incorporating solvent polarity parameters (${E}_{T}^{N}$), Stokes shift (∆ν), dipole moment changes upon excitation, and Onsager cavity radius (Δµ_B_ and a_B_) for the betaine dye. The corresponding quantities for the molecule of interest (Δµ and a) are also considered. The dipole moment (Δµ) is calculated using the slope (m) of the plot of Δν versus ${E}_{T}^{N}$, with known values of Δµ_B_ = 9.2D and a_B_ = 6 Å for betaine dye [46, 59]. The detailed data derived from these calculations are summarized in Table 4.

As a small organic molecule, JGB has rather large dipole moment values, as can be determined from the estimated values of the ground and excited states dipole moment stated in Table 4. A stronger separation of positive and negative charges within the molecule was suggested by the higher dipole moment values. Because of its high value, JGB has unique characteristics that allow it to bind to negatively charged structures, such as staining biological materials. The cationic properties of the JGB molecule and its triarylmethane structure result in a high separation of charge inside it [60].

**Table 4:** Dipole moments, slope (m) and correlation factor (r) of Janus Green B using different methods

Method	m (cm^− 1^)		µ_e_ – µ_g_	µ_g_ (D)	µ_e_ (D)
**Lippert –Matage**	1992.88		4.26	10.57	14.83
**Billot**	m_1_	35.52	0.89	13.89	14.78
	m_2_	1770.52			
**Ravi**	1125.45		1.88	10.57	12.45
**DFT**	--		3.09	10.57	13.66

### Fluorescence Enhancement of JGB by Silver nano Particles (Ag NPs)

In the realm of molecular photo physics, a current focal point is the investigation of intermolecular interactions and their impact on the spectral-luminescence characteristics of molecular systems. These interactions have the potential to induce significant alterations in the balance between radiative and non-radiative processes. Notably, silver nanoparticles (Ag NPs) play a crucial role by offering an additional decay mechanism due to their capacity for quick excitation transfer from the dye molecule [61]. Radiative or non-radiative transfer is possible for this. The presence of Ag NPs enhances the optical field of the excited fluorophore, fostering the charge transfer complexes formation. The formation of these complexes establishes an inert decay pathway for the excited fluorophore, consequently reducing its fluorescence intensity.

**Fig. 7:**
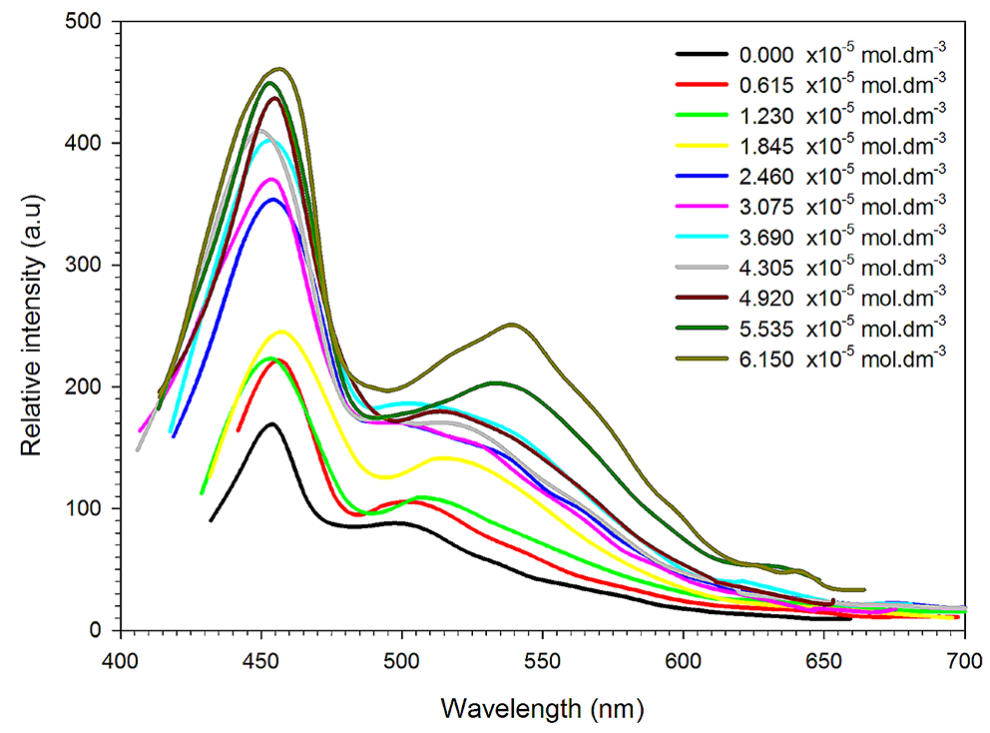
Fluorescence increasing of 2 × 10^− 5^ mol. dm^− 3^ of JGB by silver nanoparticle in water

Contrary to some studies suggesting increased resonant Raman scattering and Raman scattering in the presence of nanoparticles [62, 63], our observations reveal an intriguing phenomenon in which the fluorescence intensity increases with rising Ag NP concentration, as depicted in Fig. 7. This unexpected outcome can be attributed to two primary factors. Firstly, the “random lasing” effect, resulting from multiple light scattering on nanoparticles, prolongs the interaction period between secondary radiation photons and the active material molecules [64, 65]. In such scenarios, laser light can be generated without a resonator, with its spectrum solely determined by the spectral characteristics of the active molecules. This effect is particularly pronounced in environments containing large-sized metal NPs or nanoparticle aggregates with potent scattering characteristics.

The second contributing factor to the observed increase in fluorescence intensity is the augmented number of excited active molecules due to enhanced local optical pump fields near the NP surface. This effect is expected to manifest in active media containing NPs with discernible plasmon resonance characteristics in the employed spectrum range [66, 67]. Silver nanoparticles, exhibiting strong plasmon resonances in the visible spectrum, are noteworthy in this context. While a resonator is typically required for a laser with an equivalent medium to control the emission spectrum, Ag NPs can be employed to enhance the energy properties of active media for tunable dye lasers (DL), particularly those involving lasers on binary dye mixtures where energy transfer from donor to acceptor molecules is integral [68].

It is necessary to take into account the inner filter effect’s possible impact. Ag NPs are well known for their potent absorption and scattering abilities, especially in the visible spectrum, which can cause the fluorophores’ excitation and emission light to be attenuated. Because of the absorption or scattering of fluorescence released by the nanoparticles prior to it reaching the detector, this phenomenon may cause an underestimation of the true fluorescence intensity [65]. It’s important to recognize that the inner filter effect may change the measured fluorescence signal, even though there has been a documented rise in fluorescence intensity with growing Ag NP concentration [69, 70].

Figure 7. illustrates the fluorescence spectra of JGB in an aqueous solution with varying concentrations of silver nanoparticles (Ag NPs) at 2 × 10^− 5^ mol dm^− 3^. Interestingly, the emission intensity shows a discernible increase as the concentration of Ag NPs rises, revealing the intricate interplay between nanoparticles and the fluorescence characteristics of the active molecular system.

**Fig. 8:**
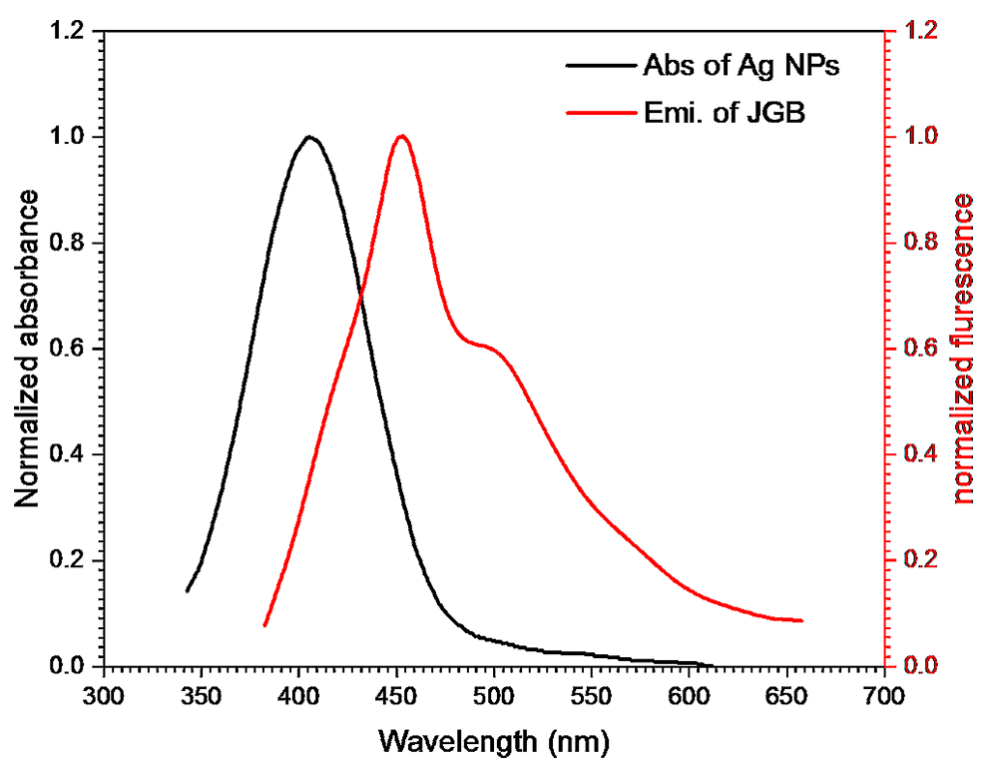
JGB’s normalized emission spectrum measures 2 × 10^− 5^ mol dm^− 3^, while Ag NPs in water have an absorption spectrum measuring 6.15 × 10^− 5^ mol dm^− 3^

The determination of critical transfer distances (R^0^) for fluorescence enhancing involves assessing the overlap between the emission spectra of the donor JGB and the absorption spectrum of Ag NPs, as depicted in Fig. 8. The substantial R^0^ values, approximately 19 nm, suggest that there exists a dipole-to-dipole interaction between the ground state Ag NPs and the excited donor JGB. This interaction results in a long-range energy transfer mechanism, which, in turn, is accompanied by the enhancing process. The high R^0^ values signify an effective dipole coupling between the donor and acceptor, indicative of a significant interaction range for energy transfer in this system [71, 72].

The thermodynamic parameter of Gibbs parameter ΔG was calculated from JGB fluorescence increasing by silver nanoparticles using the equation [73]:

$$\varDelta G=-RT\text \ {ln}\left(K\right)$$

Where R is the gas constant, T is the absolute temperature and K is the binding constant.

The binding constant K was calculated from the fluorescence spectra of JGB with different concentrations of Ag NPs in the case of fluorescence enhancement using modified Benesi–Hildebrand Eqs. [74, 75]:

$$\frac{1}{F-{F}_{min}}=\frac{1}{K\left({F}_{max}-{F}_{min}\right){\left[Ag\right]}^{NPs}}+\frac{1}{{F}_{max}-{F}_{min}}$$

Where F_min_, F, and F_max_ are the emission intensities of JGB in the absence of silver nanoparticles, at selected silver concentration, and at a concentration of complete interaction and ${\left[Ag\right]}^{NPs}$ is the concentration of silver nanoparticles.

By blotting a relation between 1/(F-F_min_) and 1/${\left[Ag\right]}^{NPs}$ the value of binding constant K was calculated from the slope and the value of F_max_ – F_min_ was calculated from the intercept. The values of the Gibbs parameter ΔG and the binding constant K were found to be 2.39 × 104 M^− 1^ and − 5951.98 cal/mol, respectively. These values indicated the thermodynamically favorable and strong binding between JGB molecules and the silver nanoparticles, suggesting a potential use of JGB as a silver nanoparticle sensor.

## Conclusion

Overall, a thorough examination of the spectroscopic and photophysical properties of Janus Green B (JGB) in various solvent environments was carried out using methods including fluorescence quenching with silver nanoparticles (Ag NPs), Time-Dependent Density Functional Theory (TD-DFT), and dipole moment analysis. These results demonstrated that solvent polarity had a significant impact on the photophysical characteristics of JGB during absorption and emission spectra. The fluorescence performance of toluene was particularly good; it achieved a quantum yield of 34% with absorption and emission cross sections of 0.8 and 9.188 cm², respectively.

Additionally, the investigation revealed higher dipole moments in the excited state relative to the ground state, suggesting a more polar excited state for JGB. Using TD-DFT simulations, the study shed light on Janus Green B’s electron density and electrostatic potential as well as the distinction between the states of highest occupied molecular orbital (HOMO) and lowest unoccupied molecular orbital (LUMO).

Furthermore, the determined K (2.39 × 10^4^ M^− 1^) and ΔG (-5951.98 cal/mol) values show a strong and advantageous interaction between JGB and silver nanoparticles, underscoring JGB’s potential as a nanoparticle sensor. This connection suggests prospects to further nanoparticle sensing technologies based on biomolecular interactions, with potential uses in biomedical diagnostics and environmental monitoring.

To sum up, our thorough investigation clarifies JGB’s possible uses in fluorescence, energy transfer and fluorescence sensing research and advances our knowledge of its behavior in a variety of solvent conditions. By using TD-DFT simulations, one may gain a comprehensive picture of the molecular behavior of Janus Green B, which is a good theoretical supplement to the experimental results.

## Data Availability

All data generated or analyzed during this study are included in this published article.
